# Design and field procedures for the clinical reappraisal of the Composite International Diagnostic Interview version 3.3 in Qatar's national mental health study

**DOI:** 10.1002/mpr.1958

**Published:** 2023-01-18

**Authors:** Iman Amro, Amal Ali, Mohamed H. M. O. Hassan, Mahmoud Al Shawwaf, Ahmed Alhassan, Dalia Al Bahari, Hana El Fakki, Zainab Hijawi, Sheeren Aly, Asmaa Amin, Rumaisa Mohammed, Marwa Nofal, Menatalla Abdelkader, Salma Salman, James Currie, Majid Alabdulla, Nancy A. Sampson, Michael First, Ronald C. Kessler, Peter W. Woodruff, Salma M. Khaled

**Affiliations:** ^1^ Social and Economic Survey Research Institute Qatar University Doha Qatar; ^2^ Hamad Medical Corporation Doha Qatar; ^3^ Department of Health Care Policy Harvard Medical School Boston Massachusetts USA; ^4^ Columbia University Department of Psychiatry New York New York USA; ^5^ University of Sheffield Sheffield UK

**Keywords:** CIDI‐WMH survey initiative, mental health, psychiatric epidemiology, Qatar, SCID‐cinical reappraisal

## Abstract

**Background:**

The Composite International Diagnostic Interview (CIDI) has been clinically reappraised in several studies conducted mainly in the US and Europe. This report describes the methodology used to conduct one of the Middle East's largest clinical reappraisal studies. The study was carried out in conjunction with the World Mental Health Qatar—the first national psychiatric epidemiological study of common mental disorders in the country. This study aimed to evaluate the diagnostic consistency of core modules of the newly translated and adapted Arabic version of the CIDI 5.0 against the independent clinical diagnoses based on the Structured Clinical Interview for DSM‐5 (SCID‐5).

**Methods:**

Telephone follow‐up interviews were administered by trained clinicians using the latest research edition of the SCID for DSM‐5. Telephone administered interviews were key in the data collection, as the study took place during the COVID‐19 pandemic.

**Results:**

Overall, within 12 months, 485 interviews were completed. The response rate was 52%. Quality control monitoring documented excellent adherence of clinical interviews to the rating protocol.

**Conclusions:**

The overall methods used in this study proved to be efficient and effective. For future research, instrument cultural adaptation within the cultural context is highly recommended.

## 1 INTRODUCTION

A growing appreciation of the global burden of mental disorders has led to numerous countries in recent years carrying out large government‐sponsored general population mental health needs assessment surveys. The early and influential Epidemiologic Catchment (ECA) study (Robins, 1981) provided the first reliable and valid methodology for conducting such surveys by using laymen‐administered fully structured research diagnostic interviews that were confirmed as clinically valid in targeted reappraisal interviews. This approach quickly became the standard and it persists to this day. Clinical reappraisal interviews are a critical part of these surveys because, unlike with many physical disorders, there are no objective tests (e.g., blood pressure, heart rate) or face‐valid self‐reports of mental disorders. Documentation of clinical validity is important to ensure relevance of results for service planning.

Clinical reappraisals provide this information by assessing the extent to which the full structured community interviews produce diagnoses consistent with independent clinical diagnoses made by trained clinical interviews in blinded re‐interviews. Clinical reappraisal studies are also important for bolstering research related to psychiatric nosology and informing the development and adoption of criteria in the DSM (American Psychiatric Association, 2013) and International Classification of Diseases (World Health Organization, 2019) systems. For example, testing of categorical versus dimensional approaches to determine whether they can differentiate between normal and pathological functioning, thereby suggesting revisions for some of the broad “Not Otherwise Specified” designation in DSM‐5 that were in DSM‐IV version (Regier, 2012).

To date, most large‐scale general population mental health needs assessment surveys have been conducted in conjunction with the WHO World Mental Health (WMH) survey initiative. Established in a collaboration between the World Health Organization (WHO) and Harvard Medical School in 1998, WMH has conducted surveys in more than 30 countries using a fully‐structured diagnostic interview known as the Composite International Diagnostic Interview (CIDI) (Kessler & Üstün, 2004). Diagnoses based on the CIDI have been shown to have good concordance with independent clinical reappraisal diagnoses based on the Structured Clinical Interview or SCID (Spitzer, 1992; Williams, 1992) in the US (Kessler et al., 1998; Wittchen, 1994) and Europe (Haro et al., 2006; Williams, 1992) as well as, most recently, in Saudi Arabia (Kessler et al., 2020).

The current report describes the design and procedures used to conduct one of the Middle East's largest clinical reappraisal studies in conjunction with the World Mental Health Qatar (WMHQ) survey, the first national psychiatric epidemiological study of common mental disorders in Qatar. Qatar is a rapidly developing nation in the Arabian Peninsula. The clinical reappraisal study aimed to evaluate the diagnostic consistency of core modules in the newly translated and adapted Arabic version of CIDI 5.0—the latest version of CIDI and the first version based on DSM‐5 criteria—against independent clinical diagnoses based on the Structured Clinical Interview for DSM‐5 (SCID‐5) (APA ‐ The Structured Clinical Interview for DSM‐5®, n.d.). We review here the challenges encountered in implementing this study and the ways these challenges were addressed.

## 2 METHODS

### 2.1 Clinical sample

WMHQ recruited a probability sample of Arabic speaking residents (Qataris and Arab expatriates) of Qatar (Khaled et al., 2021). Participants in the clinical reappraisal study had already completed the CIDI when they were contacted for a follow‐up interview. This subsample was recruited in weekly replicates using an algorithm designed to oversample respondents with CIDI diagnoses of the five core diagnostic modules in the CIDI: Major Depressive Disorder, Mania/Hypomania/Bipolar Disorder, Generalized Anxiety Disorder, Obsessive‐compulsive Disorder, and Panic Disorder. The selection algorithm selected 100% of the CIDI cases of these disorders, 25% of the subthreshold cases, and 25% of non‐cases in each replicate. These selection fractions were designed to ensure that a target of 50 cases (threshold and sub‐threshold cases combined) and 50 non‐cases would be administered the reappraisal interview for each disorder. The target numbers of 50 per disorder, in turn, were selected to achieve precision equivalent to previous studies that estimated concordance between the CIDI and the SCID interviews (Haro et al., 2006).

Weights were created to adjust for the under‐sampling of CIDI non‐cases, as failure to include such weights would lead to upward bias in estimating positive predictive value (PPV) and downward bias in estimating negative predictive value (NPV), critical operating characteristics that describe the proportion of CIDI cases that are confirmed as cases by the SCID (PPV) and the proportion of CIDI non‐cases that are confirmed as non‐cases by the SCID (NPV). A description of the sampling and weighting procedures will be presented in a forthcoming publication on clinical reappraisal study results.

A total sample of nine hundred and thirty respondents were interviewed in this study, including the pilot sample. As per the initial sample eligibility of the WMHQ study, all participants were Arab residents of Qatar at the time of interview, Qatari or Non‐Qatari, and over 18 years old.

### 2.2 Interview instrument

We used an adapted version of the Structured Clinical Interview for DSM‐5‐Research Version or SCID‐5‐RV (APA ‐ The Structured Clinical Interview for DSM‐5®, n.d.) as the clinical gold standard. The English version was first adapted by the first author of the SCID (MF) and a senior Harvard WMH team member (NS) to remove DSM‐5 criteria not utilized by the CIDI‐5 diagnostic algorithm (e.g. Criterion C for a diagnosis of Major Depressive Disorder was not operationalized) and to delete modules for disorders other than the five main assessed in the WMHQ study. This meant that diagnostic hierarchy rules were not operationalized in evaluating the CIDI diagnoses. For example, the hierarchy rule that some of the diagnoses considered in the study were not better accounted for by autism spectrum disorder was not operationalized, as this disorder was not included in the SCID diagnostic assessment for this study.

The Arabic version of Qatar's SCID began with an introductory section that covered basic socio‐demographics, past psychiatric history, health history and hospitalization overview, followed by the diagnostic modules used in the CIDI. Each diagnostic module started with screening questions that allowed the interviewer to assess participant diagnostic eligibility. Respondents who were classified by the clinical interviewer as qualifying for entry criteria (e.g. having either dysphoria or anhedonia most of the day nearly every day for 2 weeks or more) were then assessed for the full set of diagnostic criteria using the standard SCID probing and scoring approach. One noteworthy difference from standard practice, though, was that the sampling scheme enriched the reappraisal sample for CIDI non‐cases who endorsed diagnostic stem questions. In addition to allowing a more nuanced assessment of CIDI performance in distinguishing between cases and subthreshold cases, this approach also served to correct a problem that occurs in more typical clinical reappraisal studies, where only cases are over‐sampled, resulting in the great majority of respondents who endorse a diagnostic stem question being cases. Our sampling scheme made the clinical interviewer job more difficult because many of the respondents who endorsed diagnostic stem questions in our clinical reappraisal sample were, in fact, not CIDI cases.

In conjunction with this change in sampling design, we “forced” both CIDI cases and non‐cases who endorsed diagnostic stem questions into the diagnostic sections they entered in the CIDI to address the problem of respondent fatigue found in many prior clinical reappraisal studies that leads respondents who endorsed diagnostic stem questions in long initial interviews to deny the same questions in clinical reappraisal interviews. This was achieved by providing the SCID clinical interviewers with information endorsement of diagnostic stem questions in the CIDI for both CIDI cases and the enriched subsample of CIDI non‐cases who endorsed the same stem questions. Importantly, although partially unblinded to the CIDI stem question endorsement for non‐cases and cases, the SCID clinical interviewers were never informed about which respondent was a CIDI case and which a non‐case. SCID interviewers never had access to CIDI interviews, but were provided only with responses to diagnostic stem questions.

### 2.3 Translation and cultural adaptation

Researchers at Qatar University translated and adapted SCID‐5 into Arabic according to WHO guidelines (Kessler et al., 2008). Two teams (bilingual psychologist, health professional, and non‐clinical translator) independently translated the English version to Arabic. The two teams met to compare and consolidate the two versions of the translated instrument. A committee of three bilingual academics/health professionals reviewed and discussed the final Arabic translation, checking for cultural adaptation, conceptual equivalence, semantics, and CIDI concept matching. In cases of unresolved differences, an external academic professional was consulted before a final decision was made. This iterative process created a more culturally valid instrument than simple etic translation, hence back translation was not attempted.

### 2.4 Ethics

Qatar University (QU‐IRB 1219‐EA/20) and Hamad Medical Corporation (HMC MRC‐01‐19‐328) approved the study. The study's goal and methods were verbally explained to participants. Before each clinical interview, consent to participate and permission to audio‐record were verbally obtained using a phone script. All data and audiotapes were encrypted and saved on Qatar University's secure server. Each patient was assigned a case number and individual identifiers were retained in a password‐protected folder only available to the lead principle investigator, senior research assistant, and data analyst. All study researchers, including interviewers, signed confidentiality agreements preventing the sharing or use of participant personal information.

### 2.5 The SCID‐App

The SCID‐App was a mobile application developed by the IT team at the Social and Economic Survey Research Institute (SESRI) at Qatar University to support the recruitment and interviewing process. This application also linked the SCID interviewers with the study research team to monitor and track recruitment and interview activities.

The study application was updated weekly with eligible cases. Clinical interviewers could obtain case information using the fact sheet, via SCID‐App. An extracted one‐page information sheet from the CIDI interview included basic demographics, contact numbers, and key phrases endorsed by participants for each core diagnostic module of the CIDI interview.

The SCID App provided updated details about the total number of dials and the case status for each contacted case. Clinical interviewers used Cisco App to call subjects using highly secured devices (tablets) provided by the SESRI IT team. Each clinical interviewer reported the daily outcome of their working hours using the end‐of‐the‐day “daily report” function in the SCID‐App.

The SCID App was designed using the CATI lab's calling guidelines “Computer‐Assisted Telephone Interviewing (CATI)”, (Kelly, 2008). Each subject was phoned up once daily to seven times in total. Total contact attempts per day varied by number of interviewers, available cases, and working hours.

Additionally, access to the fact sheet and contact information for each case was only available through this App, which helped centralize and manage the caseload of interviewers by the research team manager. Finally, this centralization of the paradata on the survey process facilitated quality control monitoring of clinical interviewer activities.

### 2.6 Training

#### 2.6.1 Preparation

Six clinicians, including five clinical fellows and one psychiatric resident from the mental health department at HMC, were invited to the join the study as SCID interviewers. Three more medical professionals with mental health research experience were hired for 6 months to improve daily case recruitment.

The research team prepared 10 full SCID vignettes training and practicing purposes. Professor MF one of the main authors who developed the original SCID—reviewed and scored the vignettes, which were designed to illustrate threshold and subthreshold hypothetical cases for all diagnostic modules included in Qatar's Arabic version of the SCID. We pre‐assigned these vignettes to trainees to ensure coverage of multiple thresholds and subthreshold scenarios from different modules.

#### 2.6.2 Training sessions

At the beginning of December 2020, 11 professionals, including nine clinicians, the lead prinicipal investigator (LPI) and the study's supervisor received full training on administering the newly translated and adapted Qatar's version of the SCID. We conducted the training virtually via the Microsoft team in English. It spanned over 4 days, from 4 to 8 p.m.

The SCID instrument was fully explained by our expert trainer on the first day of the training program. Clinicians practiced administering and scoring the SCID while role‐playing the patient‐doctor interview scenarios using the pre‐prepared vignettes for the remainder of the program.

IT training was conducted face‐to‐face for 3 hours at SESRI—Qatar University. Tablet devices with a pre‐installed recording App and Cisco calling system were provided to all clinical interviewers. They received full training on using CATI technology, the SCID App, and the Cisco App including running and troubleshooting interviews remotely over the phone.

#### 2.6.3 Train additional interviewers

Due to attrition in the interviewers' pool, an additional three clinicians from the mental health department were trained to administer the SCID interview using the same previous training plan. The SCID training and IT training were conducted face‐to‐face over 3 days from 3 to 7 p.m. in November 2021 at SESRI‐Qatar University.

### 2.7 Team structure

**FIGURE 1:**
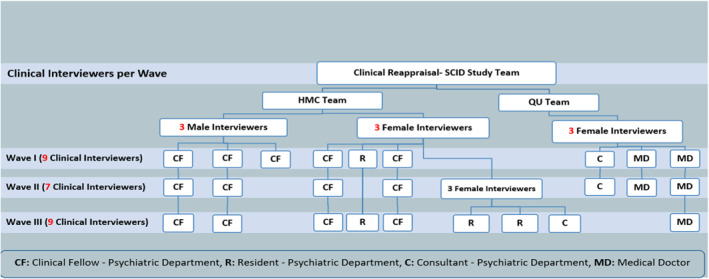
Shows the team structure of the study throughout the different waves.

Figure 1 shows the team structure of the study throughout the different waves.

### 2.8 Field operation procedures

#### 2.8.1 Pilot and study waves

The pretest sample for the month of December 2020 consisted of a total of seventy eight cases that successfully completed the CIDI and were used for piloting. Six clinicians participated in the data collection throughout the 1‐week long pilot phase, putting in anywhere from 8 to 24 h per clinician. Nineteen SCID cases were completed, with thirteen of those interviews audio recorded for quality control and monitoring purposes. At the conclusion of the pilot week, a two‐hour debriefing session was held to address all feedback and suggestions made by the clinicians in order to enhance the recruitment and data collection procedures for the study's production phase.

#### 2.8.2 Day‐to‐Day tasks

A senior research assistant trained in administering and scoring the SCID oversaw day‐to‐day tasks. The interviewers working hours were from 9 a.m. to 9 p.m., Saturday to Thursday, and 2–9 p.m. on Fridays. Clinical interviewers, study supervisor, research study team, and IT team members used “WhatsApp” to coordinate and track daily study activities. The research supervisor examined the SCID App every day for completed interviews, daily reports, and other difficulties.

The weekly timetable, number of working hours, number of completed cases, and case IDs of each completed interview per clinician were tracked daily using one Microsoft excel document for each month.

#### 2.8.3 Interview administration

The SCID interview was conducted over the phone, and responses were captured using pencil‐and‐paper. Each clinical interviewer received new hard copies of the SCID, along with the study information sheet and the consent script. The latter guided the interviewer to obtain verbal consent from the participant to run the interview and permission to audio record the interview. Hard copies of the SCID were provided to clinical interviewers periodically when needed. Hard copies of the completed SCID were collected back from the clinical interviewers every week, and data were entered manually into a unified database.

#### 2.8.4 Data collection, data management

**FIGURE 2:**
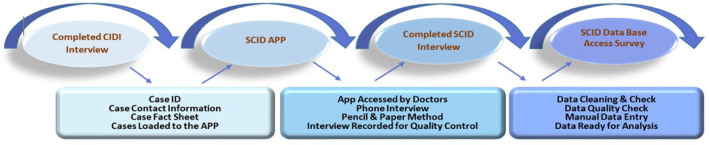
Study data collection and management procedures.

Figure 2 shows data collection process. Once the sample was loaded onto the SCID App, clinical interviewers were notified of the total new number of eligible cases per week. We sought to contact each qualifying SCID case a month after the CIDI interview. However, there were variations in the length of time between CIDI and SCID interviews. Public and statutory holidays, technical challenges, and interviewer and subject availability caused these delays, which added an extra three to 4 weeks to some cases on top of the 1‐month timetable for each completed CIDI case.

Microsoft Access 2016 was used to create the SCID Access Database (Access, 2016), which was built and further modified by Harvard research team. Data checking typically included verifying the demographics section from the SCID against corresponding CIDI information to ensure that the correct eligible case was interviewed. The data analyst verified that the skip logic and the scoring algorithm for each interviewed case were correctly executed before data entry into the Access Survey Form. In case of any discrepancy, the data analyst would contact the clinical interviewers for more details about specific cases and review the recorded interviews for data entry and scoring verification.

#### 2.8.5 Feedback and continued support

At the end of each wave of data collection, the research team conducted a debriefing session via Microsoft teams to summarize the main findings and provided feedback on the team's recruitment progress. At the end of the pilot phase, each clinician received an in‐person feedback session, which included feedback on their audio‐recorded interviews and tips on improving their performance.

#### 2.8.6 Quality control

Initial quality control checks for data included listening to the first 2–3 interviews conducted by each new interviewer. After listening to these initially completed interviews, we brought some of the interviewers back for additional training on the SCID to increase these clinicians' familiarity with the instrument and its scoring.

Bringing back some interviewers for additional training was based on one of the following conditions: (1) poor probing skills as evident from the interview recording; (2) evidence of lack of familiarity of the instrument's skip logic (e.g. scoring modules that should have not been scored based on the interviewee's responses), module‐specific scoring criteria, or overall disorder‐specific scoring on the cover sheet evident from listening to the interview recording and/or scoring of the SCID hard copies. For those interviewers, we conducted an additional training and evaluation session.

These interviewers were then given one additional mock scenario or vignette, which was pre‐scored by the two research team members, who met first to check their scores of the entire vignette and reach an agreement before meeting and checking their scores against the interviewers' own ratings of the same vignette. Discordance between the trainers and the interviewers' scores were then reviewed and discussed at length until all interviewers' understood and were satisfied with the rationale of the trainer's scoring of the vignette.

Because of the complex and lengthy nature of the SCID and re‐interviewing of community volunteers who already completed a lengthy initial interview with the CIDI, a third interview with another clinician for estimating inter‐rater reliability parameters of the SCID was not feasible. Instead, the abovementioned elaborate initial quality control checkpoint was applied to ensure validity of the scores and overall quality of the data obtained for interviewers who demonstrated initial signs of problematic scoring of cases.

After this initial quality control consolidation stage, recorded interviews were collected remotely every week from the interviewers' devices and loaded onto a secure password‐protected folder at QU University's server. The LPI, study supervisor, and data analyst only accessed the recorded interviews.

The study supervisor periodically listened to the competed interviews (up to 20% of the recorded interviews per interviewer) to ensure that the clinical interviewers adhered to all quality criteria in administering the SCID allowing the supervisor to evaluate each interviewer's performance, reduce interviewer related SCID administration errors and deviation from the original protocol. Accordingly, each interviewer received a feedback report periodically. The study supervisor kept track of all interviewers' progress and offered re‐training sessions for interviewers based on their performance.

### 2.9 Reconciliation interviews

**FIGURE 3:**
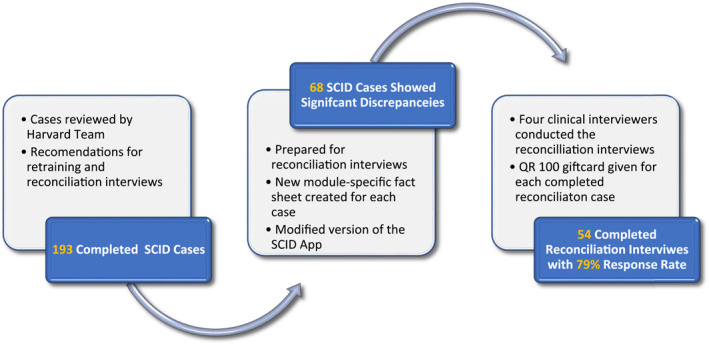
Reconciliation interviews, justification, preparation, and completion.

Upon completing the first wave of data collection, one hundred and ninety three completed SCID interviews were reviewed by the Harvard team (Figure 3). A preliminary check on the concordance and discrepancy between the CIDI and the SCID interviews was conducted. Overall, across the five diagnostic modules of interest for this study, 7% to 31% of CIDI cases were SCID non‐cases, accounting for about 55% of the total number of completed CIDI cases.

This initial finding necessitated a re‐training session for some of the clinical interviewers with high non‐case discrepancy rate where inconsistency was found high among cases reported positive for certain disorders in the CIDI interview, but negative in the SCID relative to other interviewers. The collection of this additional data aimed to improve the accuracy of the SCID interviewers so that they can generate more reliable and valid diagnoses than the CIDI interviews. The SCID‐App was modified to track the process of reconciliation interviews. The top‐performing clinical interviewers who had the least non‐case discrepancies with the CIDI exclusively conducted these interviews.

On average, the reconciliation interviews required 10 to 15 minutes to complete, using customized module‐based SCID interviews and fact sheets for each case. Subjects who completed the reconciliation interviews received an invitation SMS before contacting them; a thank you SMS note and a hundred Qatari Riyals gift card upon completion.

## 3 RESULTS

### 3.1 Selected sample and response rate

The participant recruitment phase started in February 2021 and ended in January 2022. A total of nine hundred and thirty cases were eligible to participate in the SCID study. Approximately seventy‐eight and eight hundred and fifty‐two cases were selected for the pilot and the main study production, respectively.

**FIGURE 4:**
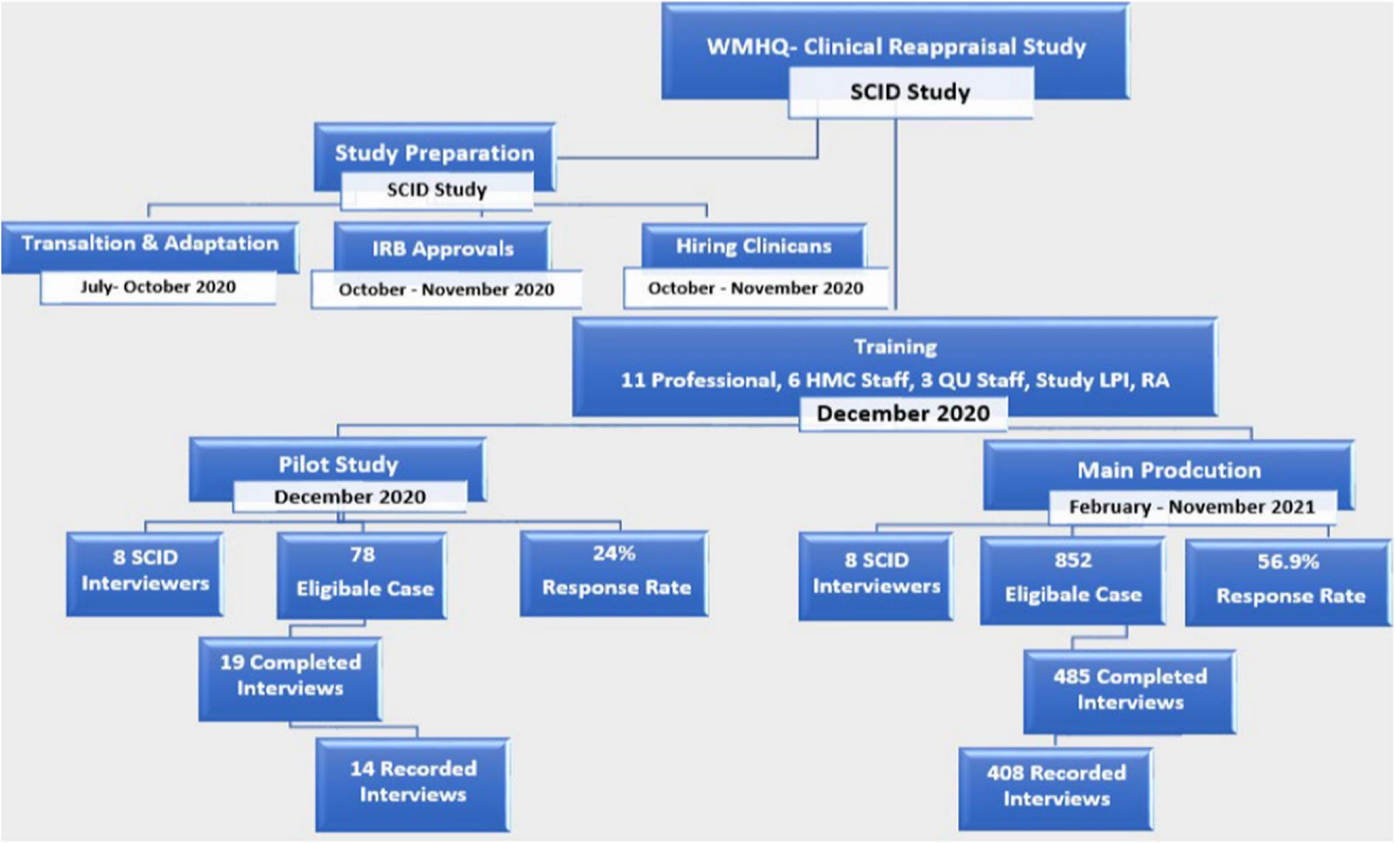
SCID study timeline, selection, completion, and response rate per phase.

Figure 4 details the study timeline and briefly describes the preparation phase for the pilot and main study. Four hundred and eighty‐five cases were completed over 13 months. The overall response rate was 24% and 52% for the pilot and main study production, respectively. On average, 90% of the eligible cases were contacted for the SCID interview within 2 weeks from the first CIDI interview completion.

### 3.2 Loaded and completed sample description

Table 1 shows the monthly number of selected samples, completed interviews, and clinical interviewers. The number of interviewers did not alter the number of monthly completed interviews. The total working hours of each interviewer and the total selected sample per month both noticeably led to an increase in the number of completed interviews at each selection. Occasionally, due to uncontacted eligible cases from the previous month, the number of completed interviews surpassed the intended sample for that month.

**TABLE 1:** Number of the sampled eligible cases completed interviews, and working interviewers per month

Study main production	Selected sample	Completed interviews *n* (%)	Total number of interviewers per month
Pilot (1 week)	78	19 (24.3)	8
Dec 2020			
Feb 2021	217	119 (54.8)	8
Mar 2021	46	49 (106.5)^a^	7
Apr 2021	17	6 (35.2)	4
Jun 2021	61	34 (55.7)	7
Jul 2021	95	15 (15.7)	1
Sep 2021	20	33 (165)^b^	5
Oct 2021	85	29 (34.1)	4
Nov 2021	182	85 (46.7)	8
Dec 2021	109	87 (79.8)	9
Jan 2022	20	19 (95)	5
Total	930	485 (52.2)	‐‐‐

^a^ Sample carried over from the previous month. However, cases were contacted within 1 month from the completion of the first interview as per study protocol.

^b^ Sample carried over from previous 2 months. However, some cases were contacted after 7–8 weeks from the completion of the first interview.

### 3.3 Sample characteristics

By January 2022, four hundred and eighty five interviews had been completed. Table 2 provides descriptive statistics for the study's participants: 79% of our sample was non‐Qatari, 60% male, and 68% married (Snoj, 2009). Nearly two‐thirds of the sample was male, averaged 38 years old and 66% of participants held a diploma degree or higher. Additionally, the table compares individuals who completed the SCID interview with those who refused among the total sample of 930 eligible cases.

**TABLE 2:** Sample characteristics for the clinical reappraisal –SCID study

Variables	Categories	Completed SCID		Did NOT complete SCID		Chi‐squared (*χ* ^2^)	Total	
		Frequency (*n)*	Percent (%)	Frequency (*n*)	Percent (%)	*p*‐value	Frequency (*n*)	Percent (%)
Gender	Male	292	60.2	224	50.3	0.002	516	55.5
	Female	193	39.8	221	49.7		414	44.5
Marital status	Never‐married	114	23.5	112	25.2	0.809	226	24.3
	Married	330	68.0	294	66.1		624	67.1
	Previously married (divorced, separated, widowed)	41	8.5	39	8.8		80	8.6
Nationality	Qatari citizen	101	20.8	161	36.2	0.000	262	28.2
	Arab nationality	384	79.2	284	63.8		668	71.8
Age group	18–24	56	11.6	72	16.2	0.144	128	13.8
	25–34	135	27.8	115	25.8		250	26.9
	35–44	164	33.8	153	34.4		317	34.1
	45–54	83	17.1	76	17.1		159	17.1
	65+	47	9.7	29	6.5		76	8.2
Education level	Secondary school or less	161	33.5	165	37.3	0.018	326	35.3
	Diploma degree	63	13.1	32	7.2		95	10.3
	Undergraduate degree	198	41.3	200	45.2		398	43.1
	Postgraduate degree	58	12.1	46	10.4		104	11.3

### 3.4 Quality report‐performance output

Our SCID interviews lasted an average of 34 min. Four hundred and eight interviews were recorded in total (see Figure 4). Audio‐recorded interviews were monitored at a rate ranging from 5% to 40% per interviewer. Following the first wave of data collection (April 2021), One hundred and ninety three SCID interviews were completed, with 35% (68 cases) revealing significant discrepancies between the CIDI and SCID interviews, indicating clear variations in case capture. We conducted reconciliation interviews in fifty‐four of the sixty‐eight cases, achieving a 79% overall response rate.

## 4 DISCUSSION

We presented here the design, techniques, and procedures for one of the largest clinical reappraisals of the CIDI to date, conducted as part of Qatar's first national epidemiological study of mental disorders—the WMHQ.

The literature search showed few published reappraisal studies within the WMH survey consortium and the field of psychiatric epidemiology in general (Kessler et al., 1998; Wittchen, 1994; Wittchen et al., 1995, 1996). Even scarcer are methodological papers describing the field operations and procedures of these types of studies conducted in the Middle East. The current study fills this methodological gap in conducting large‐scale reappraisal studies, which is important given that advances in information technology and data science make these studies more affordable to conduct at the population level than ever before.

Our study's overall response rate was 52%, which is relatively high considering the reduction of such rates in recent decades (Tuckel & O’Neill, 2002). The study results were representative of the Qatar population since non‐Qatari (male, married or previously married, highly educated) were more likely to complete the SCID interview (Snoj, 2009).

Below, we describe, the successes, pitfalls, and challenges of conducting the first methodology study in the Middle East to report on a large‐scale clinical reappraisal of the CIDI and SCID using the DSM‐5 (latest) editions in Arabic.

### 4.1 Successes

Due to the diversity of the Arabic language vocabularies, it was essential to follow the same terminologies used in the CIDI translation and adaptation phase. Therefore, we matched all terminologies with different possible translations within both instruments using the same conceptual terms and meaning.

Bilingual research team who had substantial expertise, experience, and cultural awareness of mental illness in Qatar facilitated the successful adaptation of the SCID instrument. Additionally, the dialect variations among clinical interviewers helped overcome slang and cultural disparities in Qatar's spoken Arabic.

Technological advances that facilitated conducting many study aspects remotely, like virtual training aided in reaching the target population in 13 months despite COVID‐19 pandemic restrictions. Multiple studies showed that virtual training has several advantages, including time flexibility, geographical accessibility, and cost‐effectiveness (Pinto‐Meza et al., 2005).

Our overall response rate was 52%, which was relatively high compared to previous research of this type and method (Matas‐Guiu et al., 2014; Rodriguez et al., 2006). Telephone interviews proved to be an effective and practical approach in collecting data during the COVID‐19 pandemic as it did in health research practice in general (Kempf & Remington, 2007; Rohde et al., 1997; Sobin et al., 1993) and are more cost‐effective than face‐to‐face interviews (Prescott et al., 2014). Studies that used telephone interviews to assess mental disorders were successfully validated against in‐person administration (Simon et al., 1993). Additionally, social desirability bias about sensitive topics such as mental illness are minimized compared to in‐person interviews (Al‐Habeeb et al., 2020).

The SCID App proved to be an effective tool for tracking all study activities in near real‐time. For instance, it supported using one organized platform for communication and reporting on study‐related day‐to‐day tasks, it minimized COVID‐19 cross‐contamination through indirect means by using less printed papers during the data collection phase. Moreover, the App features enabled keeping all dates and records of each case updated all the time for all APP users for easy access and tracking.

All audio recordings of the SCID interviews provided sufficient information for data quality checks and verification, and helped the study research team to settle discrepancies and differences in scoring the data for some of the SCID interviews.

### 4.2 Pitfalls

#### 4.2.1 COVID‐19 pandemic

Due to the COVID pandemic and its restrictions our options of monitoring study measures, tracking progress, and evaluating the extent of clinical interviewers' engagements during debriefing, training, and reconciliation sessions were limited.

Although the SCID App was successful in helping researchers collect data for the study, technological problems were frequent and led to unanticipated delays at several points. The availability of IT workers was occasionally insufficient, which resulted in delays in responding to technical issues and troubleshooting devices and Apps, which in turn contributed to delays in conducting the study.

Other delays in meeting the target number of completed CIDI interviews also occurred, which required time extension to the original timeline, and further arrangements to minimize the impact of such delays.

As shown in Table 1, we had a low number of qualified SCID cases during different weeks/months depending on the number of completed CIDI interviews the previous week. Response rate and SCID qualifying cases varied from week to week; this variation could have been lessened by updating the SCID sample frame more often. Due to the flexible working hours of CIDI layman interviewers, the quantity of completed CIDI interviews was also influenced, contributing to the considerable unpredictability in the number of qualified SCID cases from week to week. It is important to recognize that this resulted in the disproportionate stratified random sampling design of the clinical reappraisal study not covering the full sample, most notably under‐sampling hard‐to‐reach respondents and respondents who were surveyed late in the field period. It is conceivable that this influenced results about CIDI‐SCID concordance.

Recruiting suitable clinical interviewers to conduct the SCID interviews was one of the early challenges faced by the study. We further recruited additional interviewers who were willing to dedicate full time working hours to maximize the completed SCID cases and improve the response rate.

#### 4.2.2 SCID translation and adaptation

Due to the diversity of the Arabic language vocabularies and dialects, the clinical interviewers faced challenges related to participants' understanding of specific terminologies used during the SCID interviews. The main terminologies used in the structured portion of the SCID interview mostly matched those used in the CIDI interview; however, the unstructured part of the SCID (e.g., probes or open‐ended follow‐up questions) required deep conceptual understating of the Arabic language and its terminology among the clinical interviewers to overcome the semantic difficulties in the Arabic medical jargon when explaining to the participants (Guermazi et al., 2012). Moreover, the SCID training and practice sessions were conducted virtually in English, and the actual SCID study interviews were in Arabic. Our bilingual interviewers, who are all native Arabic speakers, faced initial challenges related to administering the SCID interviews to Arabic speakers participants only; because some of them have a limited understanding of the official Arabic language compared to slang or other specific spoken dialect of Arabic. Based on these considerations, evidence of incomplete concordance between diagnoses based on the CIDI and on the SCID in this study might reflect lack of complete validity of SCID diagnoses as much as lack of validity of CIDI diagnoses.

#### 4.2.3 Virtual training

Despite the careful consideration in putting together a successful virtual training program, ensuring clinical interviewers' engagement throughout the training days proved to be challenging due to poor Internet connection, long online training hours, less dynamic sessions, and less‐than‐ideal interpersonal interactions between the trainees.

#### 4.2.4 Fielding and data collection

Clinical interviewers had to persuade research participants to enroll in the study owing to the prolonged CIDI phone interview, which lasted 60–120 min or longer in certain cases, and the fact the CIDI interview was completed recently. Interviewers faced a large number of refusals, missed calls, and hard‐to‐reach participants who blocked the calling number or did not answer after the first contact. Clinical interviewers noted that, without body language and facial expressions, telephone interviews restricted their ability to recruit and conduct the interviews successfully.

Staying updated with all recorded SCID interviews and assessing the quality of each clinical interviewer's work was a challenge for the study supervisor. First, the research supervisor had no direct access to the devices used for data collection or recording app. The IT team needed 1 week to 1 month to extract recordings from the devices, which delayed fast input to the interviewers. Second, only one study supervisor was SCID‐trained and could monitor the quality of the recorded interviews as well as stay on track with all study activities, including daily WhatsApp communications. The lack of flexibility in the clinical interviewers' daily schedules and other commitments posed further challenges for the study supervisor, particularly when planning their weekly hours and measuring the research's success.

## 5 CONCLUSIONS

Using telephone‐administered reappraisal interviews supported with high technology‐assisted devices was an effective practical approach in the context of the pandemic. To improve the overall progress and success of this kind of study in the future, we recommend the following: First, basic instrument translation is insufficient on its own. Cultural adaption of the instrument within the local cultural context is highly recommended. Secondly, due to COVID‐19 pandemic, we were restricted in using specific methodologies in conducting this study, for example, virtual training. First‐time training should be conducted face‐to‐face and by a bilingual expert instructor. Thirdly, having sufficient IT resources including staff working during the evening and weekends is ideal. Finally, proper planning and enough trained SCID staff is needed for all study activities to minimize any issues or delays in data collection and verification.

## AUTHOR CONTRIBUTIONS

**Iman Amro:** Methodology; Supervision; Visualization; Writing – original draft. **Amal Ali:** Formal analysis; Software. **Mohamed H. M. O. Hassan:** Data collection; Interviewing the participants; Data tabulation; Reviewing. **Mahmoud Al Shawwaf:** Data curation; Project administration. **Ahmed Alhassan:** Data curation; Project administration. **Dalia Al Bahari:** Data curation; Project administration. **Hanna EL Fakki:** Data curation; Project administration. **Zainab Hijawi:** Data curation; Project administration. **Shereen Aly:** Data curation; Project administration. **Asmaa Amin:** Data collection; Interviewing the participants; Data tabulation; Reviewing. **Rumaisa Mohamed:** Data curation; Project administration. **Marwa Nofal:** Data curation; Project administration. **Menatalla Abdelkader:** Data curation; Project administration. **Salma Salman:** Data curation; Project administration. **James Currie:** Project administration; Supervision. **Majid Alabdulla:** Funding acquisition; Project administration; Resources. **Nancy A. Sampson:** Formal analysis; Writing – review & editing. **Michael First:** Conceptualization; Methodology; Visualization. **Ronald C. Kessler:** Conceptualization; Investigation; Validation; Writing – review & editing. **Peter W. Woodruff:** Conceptualization; Funding acquisition; Investigation; Project administration; Writing – review & editing. **Salma M. Khaled:** Conceptualization; Investigation; Methodology; Supervision; Writing – review & editing.

## CONFLICT OF INTEREST

In the past 3 years, Dr. Kessler was a consultant for Cambridge Health Alliance, Canandaigua VA Medical Center, Holmusk, Partners Healthcare, Inc., RallyPoint Networks, Inc., and Sage Therapeutics. He has stock options in Cerebral Inc., Mirah, PYM, and Roga Sciences.

## Data Availability

The data that support the findings of this study are available from Dr. Salma M. Khaled, the principal investigator of the study at skhaled@qu.edu.qa, upon reasonable request and pending additional ethical approval.
